# Breast Cancer in the Caribbean

**DOI:** 10.7759/cureus.17042

**Published:** 2021-08-09

**Authors:** Kristy Samaroo, Amalia Hosein, Lyronne K Olivier, Jameel Ali

**Affiliations:** 1 Biomedical Engineering, The University of Trinidad & Tobago, Port of Spain, TTO; 2 General Surgeon/Breast Surgical Oncologist, Sangre Grande General Hospital, Port of Spain, TTO; 3 Surgery, University of Toronto, Toronto, CAN; 4 Breast Unit, St. James Medical Complex, Port of Spain, TTO

**Keywords:** breast cancer management, caribbean science and public health, breast cancer mortality, breast cancer incidence, clinicopathology, breast cancer genetics, mammography usage, breast cancer control, breast cancer research

## Abstract

Breast cancer (BC) is one of the leading causes of death among women globally. In the Caribbean, there is a higher mortality rate compared with North American and European countries which have higher incidence rates. We conducted a literature review to examine the BC dynamic in the Caribbean and determine the areas where further investigations are needed. The PubMed database was used for identifying relevant studies using a combination of specific keyword searches. All studies focusing on BC within the defined Caribbean population were selected for this review. A total of 117 papers were included. The data were organized and presented under the following headings and reported according to the country where available: BC incidence and mortality, patient demographics, clinicopathology, genetics, behavioral risks, diagnosis and treatment, and BC control. Our review uncovered major variability in the incidence, management, etiology, and mortality of BC among Caribbean countries. Low-resource countries are burdened by more advanced disease with expected poorer BC outcomes (i.e., shorter periods of disease-free survival). Countries with established national cancer registries seem to have a better approach to the management of BC. The introduction of cancer treatment programs in association with international nonprofit groups has shown tremendous improvement in quality, accessible cancer care for patients, particularly in low- and middle-income settings. BC research is relatively limited in the Caribbean, lacking in both scope and consistency. The unique Caribbean BC population of diverse ethnicities, environmental influence, immigrants, socioeconomic status, and sociocultural practices allows an optimal opportunity for epidemiological investigations that can provide deeper insights into the status of BC.

## Introduction and background

**Figure 1:**
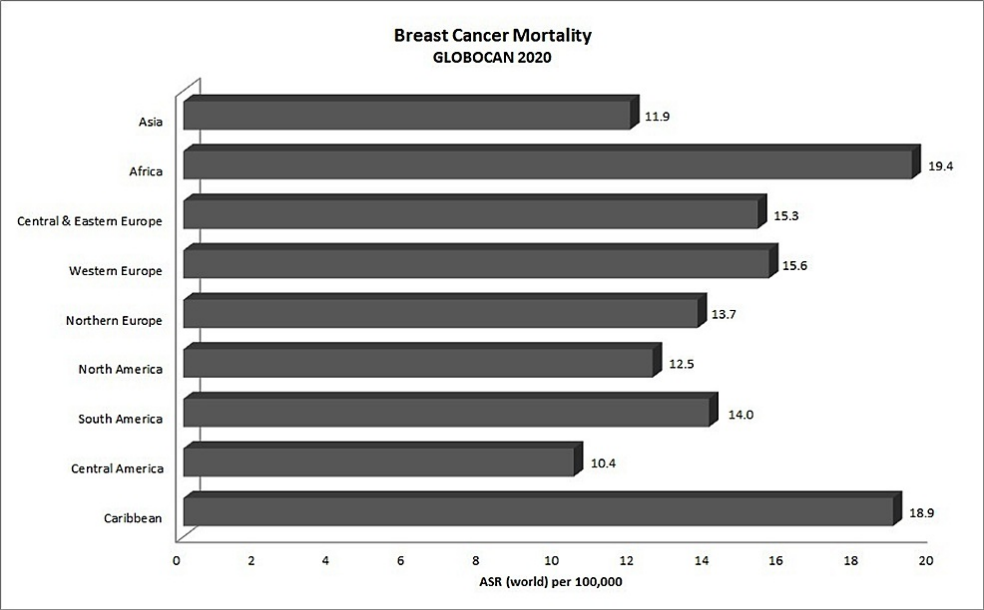
GlOBOCAN 2020 breast cancer mortality rates (ASR). GLOBOCAN: Global Cancer Observatory; ASR: age-standardized rates

**Figure 2:**
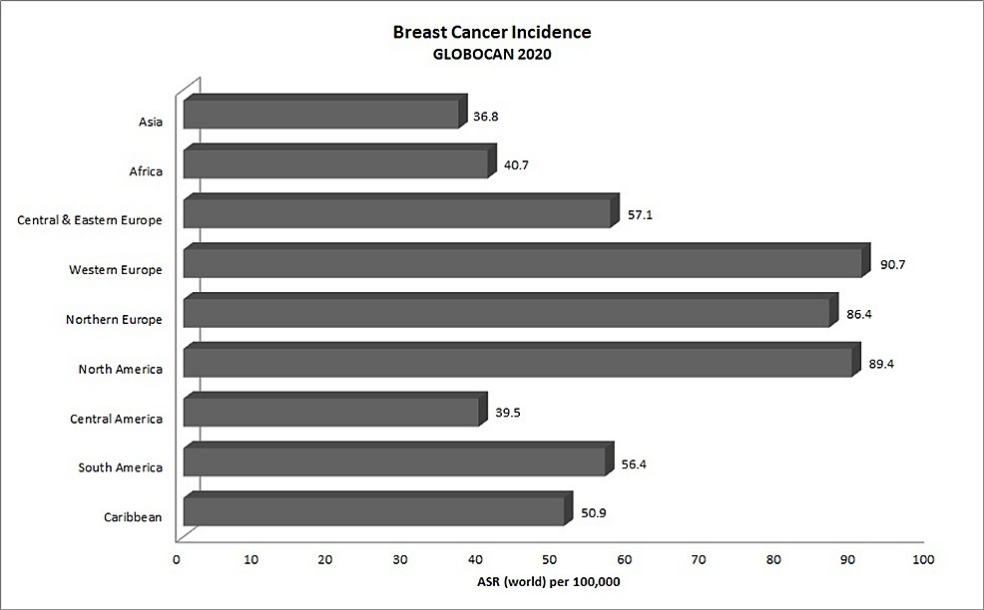
GLOBOCAN 2020 breast cancer incidence rates (ASR). GLOBOCAN: Global Cancer Observatory; ASR: age-standardized rates

Breast cancer (BC) has remained the foremost cause of cancer death affecting women globally and has now risen to the top, surpassing lung cancer, with 2.3 million new cases reported as of 2020 since its discontinuous rise in incidence over the years [1,2]. GLOBOCAN 2020 estimates reported approximately 685,000 BC deaths globally in 2020 [3]. In the Caribbean, BC, with 15,000 new cases in 2020 and a five-year prevalence of 219 per 100,000 [3], has been the second leading cancer after prostate cancer for the last two decades [4]. For the decade 2003-2013, BC was the leading cause of cancer death among women in 21 out of the 23 Caribbean territories reported, with cervical cancer being the leading cause in the other two countries and BC the second-highest following prostate cancer [5]. Although BC is considered to be a disease that affects only women, incidence among men has been rising with relatively higher mortality compared to BC in women [1], which may be due to delayed diagnosis owing to its rarity in men. Additionally, higher BC age-standardized mortality rates (AS-MR) have been reported from the Caribbean compared to those reported from North America and Europe, even though the Caribbean has comparably lower age-standardized incidence rates (AS-IR) (Figures 1, 2) [3]. This is also seen in South America, which lies within the same geographical region as the Caribbean, which has similar incidence rates, yet the Caribbean suffers higher mortality comparable to that of Africa. As the Caribbean Community (CARICOM) has approximately 83% Afro-Caribbean people (6% East Indian, 9% mixed, and 2% others) [6], investigations into genetic and ethnic predisposition of BC need to be studied. Additionally, further epidemiological investigations into the factors associated with BC outcomes observed in the Caribbean population are imperative for improving survivorship.

A systematic review of BC research in the Caribbean conducted in 2017 found the existing literature to be limited and showed the need for stronger studies, focused on treatment regimens, socioeconomic impact, environmental and behavioral factors (compliance to health screening strategies), as well as livelihoods of BC survivors [7]. These factors can provide more reliable information for the management of this disease. To further support this, another systematic review concluded that study quantity, quality, and variability in outcomes and reporting restricted the synthesis of evidence on the role of social determinants on BC in the Caribbean [8]. Globally, further investigations into this major health issue are necessary as there is no definitive consensus on the precise risk factors and protective factors related to this burdensome disease [1]. This literature review aims to examine the BC dynamic in the Caribbean to highlight where further investigations are needed.

## Review

Methodology

**Figure 3:**
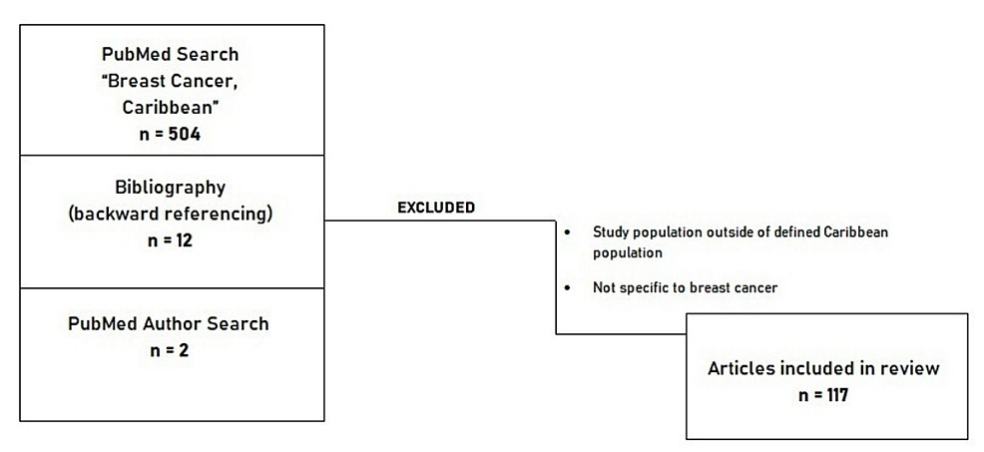
Flowchart of the search strategy for article selection.

This review included all available publications within the last 63 years (1958 to April 2021) resulting from the key search words “Breast Cancer and Caribbean” on the PubMed database. The bibliographies of several articles were also searched to identify additional publications that may not have been available on PubMed search results. Key authors identified were also searched on PubMed to retrieve any further publications that may have been missed. Abstracts and full texts were reviewed for inclusion eligibility. All studies focused on BC within the defined Caribbean population (Appendices) were included, as depicted in Figure 3. Studies comparing Caribbean data with those in other populations of different geographic regions or with immigrant Caribbean populations were also included. Finally, a total of 117 articles that met the inclusion criteria were included in this review.

The data were organized and presented under the following headings and reported by country as the available literature permitted: BC incidence and mortality, patient demographics, clinicopathology, genetics, behavioral risks, diagnosis and treatment, and BC control.

From each publication, study sample size, number of publications by country, geographic coverage, and important findings by topics discussed were examined and reported under the following headings: BC incidence and mortality, patient demographics, clinicopathology, genetics, behavioral risks, diagnosis and treatment, and BC control. GLOBOCAN 2020 was used for BC incidence and mortality for comparing all regions discussed. All incidence and mortality rates are reported as age-standardized rates (ASR) to the global population (ASR per 100,000) unless otherwise specified.

We examined seven themes, namely, BC incidence and mortality, patient demographics, clinicopathology, genetics, behavioral risks, diagnosis and treatment, and BC control for 31 Caribbean territories, which are listed and profiled in Table 1. More territories had gaps in the literature based on the themes than was expected, with seven territories without published information on any of the themes, as shown in Table 2.

**Table 1:** Profiles of each Caribbean country included in this review. Sources: Pan American Health Organization, United Nations Human Development Index, World Bank, Worldometer.info (Population Statistics).

Caribbean territory	Location	Territory	Population (2020)	Male-to-female ratio (2018)	Ethnic composition	Income classification	GDP per capita (US$ 2019)	Human Development Index (HDI 2019)	Adult literacy (%)	Female mean life expectancy at birth (2018)	Male mean life expectancy at birth (2018)	Mean life expectancy at birth for the total population (2018)
Anguilla	Northern Caribbean	United Kingdom	15,003	83:91	85.3% African 4.9% Hispanics	-	-	-	93	84.3	79	81.6
Antigua and Barbuda	Northern Caribbean	Independent	97,929	50:54	90% African	High income	$17,112.80	0.778	98.4	74.2	79	76.7
Aruba (Dutch Caribbean)	Southern Caribbean	Independent (The Netherlands)	106,766	25:28	Mixed: Dutch Caribbean and The Netherlands	High income	$29,007.70	-	99.4	73.6	78.5	76.2
Bahamas	Northern Caribbean	Independent	393,244	49:51	85% African 12% Caucasian 3% Hispanic/Asian	High income	$32,863.70	0.814	-	72.9	78.9	76
Barbados	Eastern Caribbean	Independent	287,375	137:149	>90% African	High income	$18,148.20	0.814	97	73.8	78.5	76.2
Belize	Central America	Independent	397,628	95:96	52.9% Mestizo 26% Creole 6% Garifuna 11% Indigenous Mayans	Upper-middle income	$4,815.20	0.716	80	68.1	73.8	70.8
Bermuda	Northern Caribbean	United Kingdom	62,278	34:37	54% African 31% Caucasian 7.5% Mixed	High income	$117,089.30	-	-	78.3	84.7	81.5
Bonaire (Dutch Caribbean)	Western Caribbean	The Netherland Antilles	20,900	-	Mostly Dutch	-	-	-	-	-	-	-
British Virgin Islands	Northern Caribbean	United Kingdom	30,231	1:1	Majority African	High income	-	-	97.9			78.9
Cayman Islands	Western Caribbean	United Kingdom	65,722	29:31	20% African 20% Caucasian 40% Mixed	High income	$85,975.00	-	-	78.7	84.2	81.4
Cuba	Western Caribbean	Independent	11,326,616	43:46	9.3% African Cuban 64% European Cuban 27% Mulatto/Mixed	Upper-middle income	$8,821.80	0.783	-	78.2	82.1	80.1
Curacao (Dutch Caribbean)	Southern Caribbean	Independent (The Netherlands)	164,093	37:44	Majority African Dutch/Dutch Caribbean	High income	$19,689.10	-	-	75.6	81.5	78.7
Dominica	Eastern Caribbean	Independent	71,986	1:1	85% African 4% Indigenous Kalinago 1.5% Caucasian	Upper-middle income	$8,110.60	0.742	-	74.4	80.5	77.4
Dominican Republic	Northern Caribbean	Independent	10,847,910	17:66	11% African 16% Caucasian 72% Mulatto/Mixed	Upper-middle income	$8,282.10	0.756	90.9	71.2	77.5	74.2
French Guiana	South America	France	298,682	1:1	Mix of Indigenous, Europeans and Africans	-	-	-	-	77.4	83.5	80.4
Grenada	Southern Caribbean	Independent	112,523	1:1	82% African 2% East Indian 13% Mixed	Upper-middle income	$10,808.70	0.779	98	71.5	76.4	73.9
Guadeloupe	Eastern Caribbean	France	400,124	104:120.5	75% African/Mulatto 11% Caucasian 9% East Indian 2% Lebanese/Syrian	-	-	-	-	78.3	84.9	81.8
Guyana	South America	Independent	786,552	95:87	26% African 40% East Indian 11% Indigenous 20% Mixed	Upper-middle income	$6,609.60	0.682	85	64.6	69.3	66.9
Haiti	Northern Caribbean	Independent	11,402,528	123.5:154.75	95% African 5% Caucasian/Mulatto	Low income	$1,272.50	0.510	76.5	61.6	66.1	63.8
Jamaica	Northern Caribbean	Independent	2,961,167	14:19	90% African	Upper-middle income	$5,582.30	0.734	91.7	73.9	78.6	76.2
Martinique	Eastern Caribbean	France	375,265	5:6	90% African/Mixed 5% Caucasian 5% East Indian/Chinese	-	-	-	-	79.3	85.3	82.4
Montserrat	Eastern Caribbean	United Kingdom	4,992	1:1	88% African 3% Hispanics 3% Mixed	-	-	-	-	76.1	73.5	74.8
Saba (Dutch Caribbean)	Northern Caribbean	The Netherland Antilles	2,000	-	Mostly Dutch	-	-	-	-			-
St. Kitts and Nevis	Eastern Caribbean	Independent	53,199	1:1	90% African 3% Caucasian 2% Mixed	High income	$19,935.00	0.779	97	73.7	78.7	76.2
St. Lucia	Eastern Caribbean	Independent	183,627	22:23	85% African 2% East Indian 11% Mixed <1% Indigenous	Upper-middle income	$11,611.40	0.759	99.3	73.2	78.6	75.9
St. Vincent and the Grenadines	Eastern Caribbean	Independent	110,940	56:55	71% African 23% Mixed 3% Indigenous 1% East Indian	Upper-middle income	$7,457.50	0.738	70.1	71.3	75.7	73.4
Sint Eustatius (Dutch Caribbean)	Northern Caribbean	Netherland Antilles	3,140	-	Mostly Dutch	-	-	-	-	-	-	-
Sint Maarten (Dutch Caribbean)	Northern Caribbean	Independent (The Netherlands	42,876	21:22	-	High income	-	-	-	76.1	80.9	78.5
Suriname	South America	Independent (The Netherlands	586,632	85:83	27% Indian 22% Maroon 16% Creole 14% Javanese 13% Mixed 4% Indigenous	Upper-middle income	$6,359.80	0.738	94.4	68.5	75	71.6
Trinidad & Tobago	Southern Caribbean	Independent	1,399,488	76:97	34% African 35% East Indian 23% Mixed 8% Other	High income	$17,398.00	0.796	98.7	67.4	74.6	70.9
Turks and Caicos Islands	Northern Caribbean	United Kingdom	38,717	15:14	35% Haitian Immigrants 8% Jamaicans 5% Dominican Republic	High income	$31,353.30	-	98	77.3	83	80.1

**Table 2:** Coverage of information available from review articles for each Caribbean territory.

Caribbean territory	No. of articles	Incidence	Mortality	Patient demographics	Clinipathology	Behavioral risks	Genetics	Control	Treatment	Other
Anguilla	0									
Antigua and Barbuda	4	X	X	X						
Aruba (Dutch Caribbean)	2	X	X	X		X				X
The Bahamas	9	X	X	X	X	X	X	X	X	X
Barbados	6	X	X	X	X	X	X	X	X	X
Belize	0									
Bermuda	1	X		X						
Bonaire (Dutch Caribbean)	1	X		X						
The British Virgin Islands	0									
Cayman Islands	1			X	X	X	X			
Cuba	8	X	X	X		X	X	X		X
Curacao (Dutch Caribbean)	2	X	X	X		X				X
Dominica	1			X	X	X	X			
Dominican Republic	0									
French Guiana	1	X	X	X	X			X	X	
Grenada	3	X	X	X		X		X		
Guadeloupe	5	X	X	X	X	X		X		
Guyana	1			X	X				X	X
Haiti	11	X	X	X	X	X		X	X	X
Jamaica	17	X	X	X	X	X	X	X	X	X
Martinique	6	X	X	X		X		X		
Montserrat	0									
Saba (Dutch Caribbean)	1	X		X						
St. Kitts and Nevis	1		X	X		X		X		
St. Lucia	0									
St. Vincent and the Grenadines	2	X	X	X		X		X		
Sint Eustatius (Dutch Caribbean)	1	X		X						
Sint Maarten (Dutch Caribbean)	1	X		X						
Suriname	2	X		X	X				X	X
Trinidad and Tobago	25	X	X	X	X	X	X	X	X	X
Turks and Caicos Islands	0									
Multiple Regions, Not Specified	18	X	X	X	X	X	X	X	X	X
All	2	X	X	X		X		X	X	

Breast cancer incidence and mortality

Only four countries reported high-quality incidence data with coverage of 14.4% of the Caribbean population while eight other countries had established national cancer registries [9,10]. Therefore, assessing regional BC incidence and mortality estimates is challenging for the Caribbean region [10].

Antigua & Barbuda

Of the 492 histologically confirmed new cancer cases for the five-year period from 2001 to 2005, 76 were for BC with an incidence rate of 37.6 per 100,000 [11]. The same period recorded 354 cancer deaths, of which 45 (30% of female cancer deaths) were from BC at a mean age of 60.6 years, with a mortality rate of 21.79 per 100,000 [12]. This study revealed a 4% increase in BC cancer mortality among women over their 1984-1989 study [12].

The Bahamas

The cumulative incidences of BC for the years 2009-2011 were 51.4, 45.4, and 51.4, respectively (~50 per 100,000 cumulative incidence rate), with the highest incidence among women aged 40-59 years [13]. By comparison of the Human Development Indexes (HDIs), The Bahamas, with an HDI of 0.789 for BC, had the third-highest incidence rate among all the countries in this range, ranking higher than larger Caribbean countries such as Jamaica (HDI: 0.715; incidence rate: 42) and Trinidad and Tobago (HDI: 0.776; incidence rate: 48) [13]. In Grand Bahama Island, the annual age-standardized cancer mortality rate was 114.8 per 100 000, with BC accounting for 19.2% of cases, and 45.3% of all cases out of the 167.7 per 100,000 age-standardized cancer incidence rate for the 15-year study period [14].

Barbados

The Pan American Health Organization (PAHO) estimated AS-MR at 22.1 per 100,000 and AS-IR at 94.7 per 100,000 in 2016, the highest in the Caribbean along with The Bahamas, where mortality rates were approximately two times as high as those in Canada and the United States (15.92 compared to 9.17 and 9.27, respectively, according to GLOBOCAN 2018 estimates) [3,15]. Over a decade earlier, the AS-MR in Barbados was 32.9 (29.9-36.0) per 100,000 for the period 2002-2006, similar to those reported in the United States at the time. However, Barbadian AS-IR at 58.4 per 100,000 was comparably lower than that of African American women at 104.9 per 100,000 [16]. Hercules et al. [17], reported between 160 and 251 total annual cases from January 2007 to December 2016, similar to Trinidadian trends.

Bermuda

The Bermuda Cancer Registry for 2000-2003 showed an age-adjusted BC incidence rate of 145 per 100,000 black women (142.6 crude rate) compared to 169.2 per 100,000 white women (161.3 crude rate), a ratio of 0.73 (0.56-0.94; p < 0.05) [18]. As no race-specific data were available for Bermuda, the overall age-adjusted (according to the 2000 U.S. standard population by five-year age groups) mortality rate was estimated to be 49.2 per 100,000 Bermudan women (45.7 crude rate) [18].

Cuba

BC is reportedly the most common malignancy in women in all but one Cuban province with rising incidence since 1986 [19,20], despite having a national screening program. The published incidence of BC in Cuba was 40 per 100,000 per year according to the Cuban National Cancer Registry in 2008 [21], currently estimated at AS-IR of 44.3 and AS-MR of 12.9 according to GLOBOCAN 2020 [3]. This shows a continuous rise in incidence over Cuban history with 24.4 per 100,000 in 1977 to 31.4 per 100,000 in 2000 [22]. Boschmonar et al. [23] found BC survival rates among Cuban women to be comparable to African Americans and Europeans.

Dominica

Razzaghi et al. [5] reported 52 BC deaths in Dominican women during the decade 2003-2013, which was the leading cause of cancer death among women. The PAHO reported a BC AS-MR of 16.4 per 100,000 in 2013 [15]. No data were found for BC incidence.

French West Indies (French Guiana, Guadeloupe, and Martinique)

The incidence of BC in the French West Indies averaged 59.4 from 2008 to 2011, which increased from 32.8 to 45.2 per 100,000 women during 1999-2006 [24]. AS-IRs for BC were considerably lower in the French West Indies than among African Americans, European Americans, and Mainland France during the same period [25]. Data from the cancer registry of Guadeloupe for the period 2008-2013 presented incidence and mortality rates for BC at 71.9 per 100,000 and 14.1 per 100,000 respectively; age-specific incidence rates were comparable to European and U.S. populations below the age of 45, higher in Guadeloupian women aged between 45 and 55 years [26]. The doubling of the incidence rate of BC from 37.2 in previous years (1999-2006) provides strong evidence of the need for improved cancer management and control [25]. An increase from 6,832 cases of cancer in Martinique women during the 20-year period from 1981 to 2000 to 8,938 for the period 2001-2015, with a subsequent rise in BC as the leading female cancer from 1,568 cases (22.95% of female cancers) to 2,873 cases (32.14% of female cancers), respectively, giving rise to an upward trend of the incidence rate of BC from 35.8 per 100,000 to 65.9 per 100,000 women [27,28]. With 226 new cancer cases per year and 58 deaths per year, BC is the prime tumor site in Martinique women in terms of incidence and mortality with a mortality rate of 14.4 per 100,000 for 2011 to 2015, analogous to Guadeloupe [28]. In French Guiana, AS-IR was 47.1 per 100,000 women and AS-MR was 11 per 100,000 women from 2003 to 2006 [29].

Grenada

For the 10-year period from 2000 to 2009, there were 153 new female invasive BC cases accompanied by 89 deaths, comprising 17% of all female cancer deaths with a mortality rate of 17.7 per 100,000 and an observed 30% increase in mortality (p < 0.05) over the period [30]. The incidence rate for BC at 32.9 per 100,000, the most prevalent of female cancers, was half the incidence rate for prostate cancer, the most prevalent cancer overall [30]. This shows a decrease in incident BC cases from the decade prior (49.1 per 100,000) and a relatively stable mortality rate for BC of 17.9 [31]. Razzaghi et al. [5] later reported an increase to 25.5 per 100,000 five-year AS-MR for BC from 2003 to 2013.

Haiti

DeGennaro et al. [32] estimated the incidence of BC in Haiti to be 23.9 per 100,000 women with a prevalence of 88.4 per 100,000 women according to GLOBOCAN 2012, and an approximate mortality rate of 45%. In 2018, the estimated number of new BC cases was >1,100, with a mortality-to-incidence ratio of >60% [33].

Jamaica

From 1998 to 2007, BC cases accounted for approximately 28% of all female cancers in Kingston and St. Andrews, Jamaica [34,35]. The highest proportion (~55%) of this BC population was women between the ages of 25 and 59 years [34-36]. Between 2010 and 2014, a total of 1,634 Jamaican women died of BC, accounting for 24% of all female cancer deaths [36]. While the overall mortality rate during this period was not statistically significant, the AS-MR for BC steadily increased from 21.8 in 2010 to 28 in 2014 for the entire female population. Moreover, an analysis of the five-year mortality trend reflected by the annual percentage rate of change per 100,000 showed a statistically significant increasing tendency (p = 0.028) [36]. Of the many reasons that could attribute to the increasing BC mortality rates, the highest mortality observed among the ≥75-year age group is a public health concern with the growing elderly population and limited clinical management offered to these BC patients [36].

The Netherlands Antilles (Aruba, Bonaire, Curaçao, Saba, St. Eustatius, and Sint Maarten)

A 1981 study seeking to establish a national cancer registry reported 164 cases per 100,000 for females and a global standardized incidence rate for BC of 41.2, by far the most frequent malignant lesion in females and leading cancer for the 12-year study period from 1968 to 1979 [37]. This rate was among the highest in the tropical belt, surpassing Cuba (28.0 per 100,000) and Jamaica (39.6 per 100,000) at the time [37]. Verstraeten et al. [38] reported a higher mortality-to-incidence ratio in Aruba and Curacao (0.29 and 0.28, respectively) than in The Netherlands (0.21) perhaps as a result of lower levels of BC screening in the Dutch Caribbean.

Saint Kitts and Nevis

In Nevis, the smaller of the twin-island nation, crude BC mortality rate declined from 27 per 100,000 women in 2002 to 18 in 2003, further declining by 50% to nine deaths per 100,000 females from 2004 to 2006 [39]. Age at death was unavailable to calculate the age-adjusted mortality rate. Felix et al. [39] suggested that these rates may be underestimated as Nevisian women typically travel overseas for BC care.

Saint Vincent and Grenadines

A total of 110 (106 females, 4 males) new cases of BC were identified during the period 2007-2011 [40,41]. Of the 4,197 deaths recorded between 2008 and 2012, approximately 10% (n = 62) were due to BC [40,41].

Suriname

From 1980 through 2000, BC was the third most common malignancy overall and the second most common cancer in women [42]. Annual numbers of new BC cases were 17 per 100,000 women [42]. van Leeuwaarde et al. [43] calculated the age-adjusted incidence rate to be 28 per 100,000, which is less than half that seen in first-generation Surinamese women in The Netherlands at 65 per 100,000. They also found the median incidence to be 38.5 cases per year, with a peak of 70 BC patients in 2000 [43].

Trinidad and Tobago

There are 250 new cases of BC reported on average annually with 125 deaths per year [44]. It is suggested that for every two women with BC, one may die (incidence:death ratio of 2:1) [45]. An analysis of the national cancer registry for the period 1995-2009 showed incidence and mortality rates for BC to be 46.6 and 18.4, respectively, which are among the highest in the region [46] and comparably higher than the global trends. The annual cumulative incidence rate of BC, as determined for the calendar years 2010 and 2011, in north Trinidad was 32.4 per 100,000 and 24.6 per 100,000, respectively [47]. Ethnic and geographic disparities in incidence and mortality rates also exist with the majority of cases (~53%) occurring in women of African ancestry and among those living in urban, densely populated areas and marginally higher mortality in women of Indian descent compared to those of African descent, also observed in Guyana [48,49]. The five-year BC survival for the period 1995-2007 was between 24% and 38% and 10-year survival was between 5% and 8% [48]. Before this, from 1970 to 2004, a general pattern of increase was observed in both crude and AS-MR; the overall average crude mortality was 15.6 per 100,000 women (95% confidence interval (CI): 13.9-17.1) and the average AS-MR was 18.0 per 100,000 women (95% CI: 16.7-19.2) [50]. There was a pattern of increase in mortality with increasing age that was considerably higher (two-thirds) for the age groups above 50 years than those less than 50 years of age; however, both showed an upward trend over the 35-year period [50].

Summary

**Figure 4:**
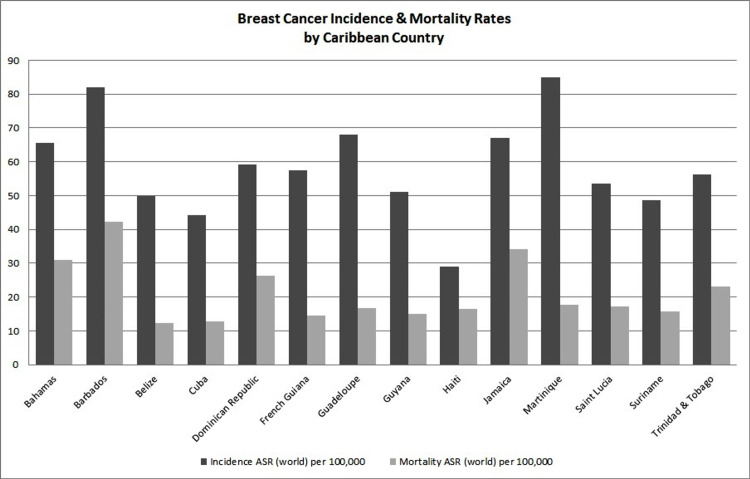
Breast cancer incidence and mortality rates by Caribbean country. Data sources: GLOBOCAN 2020 and IACR 2021. ASR: age-standardized rates

The highest BC incidence in the Caribbean was seen in Barbados, followed by The Bahamas, with the lowest incidence in Haiti. However, the most recent BC statistics available, as seen in Figure 4 which represents GLOBOCAN 2020 estimates for the available Caribbean countries, Martinique is positioned in the lead followed by Barbados and Guadeloupe. Barbados not only has the highest mortality rate in the Caribbean but the highest in the world [51]. This contrast in data provides evidence in support of the need for high-quality standardized cancer registries for each Caribbean territory.

Patient demographics

The World Health Organization (WHO) correlates the increasing incidence of BC in developing countries with increased life expectancy, urbanization, and adoption of western lifestyles, combined with a lack of access to adequate comprehensive breast care resulting in higher mortality in the Caribbean [48,52]. This burden is also attributable to the upsurge in the number of women with major BC risk factors, including lower age at menarche, late age at first pregnancy, fewer pregnancies, shorter or no periods of breastfeeding, and later menopause, in addition to rising obesity, alcohol consumption, inactivity, and use of hormone replacement therapy [52]. The most widely studied cancer patient demographics include age at diagnosis, ethnicity and socioeconomic status, as well as specific behavioral risks.

The Bahamas

The Bahamas is a Caribbean island country (an archipelago of 700 islands, of which only 28 are inhabited) with a total population of approximately 392,000, made up of 85% African origin, 12% Caucasian origin, and 3% Asian and Hispanic origin [13,15]. The female population between the ages of 30 and 69 years is 99,960, of which approximately 100-150 new cases of BC are recorded per year, typically at the average age of 42 with 50% diagnosed premenopausal [13,15,53]. However, another study found the mean age at diagnosis to be 56.6 ± 13.8 years with predictable peak cases among women of African origin (prominent ancestry), with the highest occurrence on the island of New Providence among all the islands of The Bahamas [13]. Interestingly, the incidence is higher in urban areas populated by people of lower socioeconomic status compared with people of higher social status living in nonurban settings [13].

Barbados

Barbados is an independent Caribbean island nation in the western Atlantic Ocean, with an estimated population of about 285,000 where over 31 are of African descent, approximately 4% of European origin, while South Asian and other ethnic groups account for less than 2% of the population [16]. African Barbadians and African Americans share a common ancestry, having descended from the very select subgroup who survived the long ocean voyage from West Africa during the diaspora [54]. In contrast to other Caribbean islands, Barbados had no indigenous population, remained fairly homogeneous, and had limited admixture over time, making their population an ideal subgroup of the African diaspora for epidemiological studies regarding environmental, genetic, and cultural influences on BC [54]. The Barbadian female population was estimated to be 144,803 (52% of the resident population) in 2010, with 78,090 women between the ages of 30 and 69 years in 2016 [15,17]. The Barbados National Cancer Study (BNCS) is a nationwide case-control study investigating environmental and genetic factors for BC in a predominantly African-origin population with similar ancestry as African Americans [54].

A significant increase in reported ASR-IR for BC was observed from 2009 at 78.6 per 100,000 to 135.1 per 100,000 by 2020 [16,17]. According to Hennis et al. [16], incidence peaked at 226.6 (174.5-289.4) per 100,000 among Barbadian women aged 50-54 years, and declined thereafter, a pattern in marked contrast to trends in African American women, whose rates continued to increase to a peak of 483.5 per 100,000 in those aged 75-79 years. While there was no statistical difference observed for ≤39 years, marginal statistical differences existed among women aged 40-54 years and strongly significant differences among women aged ≥ 55 years (p ≤ 0.001 at all older ages) [16]. Findings from the BNCS observed 37% of cases were aged ≤50 years and 45.5% were beyond stage I of disease at the time of diagnosis [54]. While Hercules et al. [17] reported the lowest (110.5 per 100,000) and highest incidence rates (171.9 per 100,000) in 2015 and 2008, respectively.

Cuba

Cuba is an island nation of 11 million inhabitants, divided into 169 municipalities, with a female population of 33,200 on average per municipality in 2001 [19]. Approximately 2,400 new cases of BC are diagnosed annually in Cuba [21], a 37% increase from two decades prior [20]. The Cuban National Cancer Registry reported alarming BC incidence rates for women between 35 and 55 years; 36 per 100,000 aged 35-44 years and 71.89 per 100,000 aged 45-54 years. Overall, for all ages, AS-IR was 33.06 and AS-MR was 14.58 during 1986-1990 [20]. Though the Cuban registry reports the highest BC incidence in Havana City (second highest incidence of primary cancers), which is expected given it is the country’s capital and the most populated province (~2 million inhabitants), one study identified a high-risk BC region (conditional autoregressive-smoothed relative risk [RR] between 1.21 and 1.26) in municipalities of Ciudad de La Habana province, which is a highly urbanized, predominantly white population (historically related to socioeconomic status) with late age at first birth and low parity [19].

Dominica

Dominica is an eastern Caribbean island with a population of about 70,000, of which 14,000 are women between the ages of 30 and 79 years [15]. Dominica is also a predominantly black population where BC is the leading cause of death in females, accounting for 22.5% of cancer deaths in women from 2003 to 2013 [5].

French West Indies

Guadeloupe is a French overseas Caribbean archipelago with a population of about 404,000 inhabitants at a mean age of 37 years (2009 census) and ethnically populated by 80% African-descent, 15% Indian-descent, and 5% European-descent individuals [26]. Martinique is also a French overseas department, a Caribbean island with 381,500 inhabitants of predominantly African ancestry [27]. These two islands form a major part of the French West Indies, characterized by a lower median income, larger income inequalities, a lower educational level, and a higher rate of unemployment [55]. Women living in the most deprived areas of the French West Indies had a nonsignificant higher incidence of BC (RR: 1.15; CI: 0.90-1.45) [55]. French Guiana has a relatively better socioeconomic status, resulting in a 30% immigrant population (from the Caribbean and South America) and supported by the healthcare system from mainland France [29]. The global age-standardized incidences for invasive BC calculated by summing the number of expected cases for each five-year age group found that the French West Indies populations were characterized by roughly symmetric distributions, with the largest numbers of expected cases occurring in women between the ages of 45 and 54, whereas the largest numbers of expected cases from African American and European American women from the United States and the population from mainland France occurred in women between the ages of 45 and 69 with a bimodal distribution [25]. Roue et al. [29] also reported the highest incidence among women aged 45-54 years in French Guiana.

Grenada

Grenada is a tri-island state (Grenada, Carriacou, and Petit Martinique) in the Eastern Caribbean with a population of 107,000, of which the female population comprises 21,293 (aged 30-69 years), and is typically of African descent (80%), with a mix of East Indian, and European descent [56]. BC is the leading cause of death in the predominantly black female population in this country [30]. Little is known about specific cancer patterns because Grenada does not yet have a functioning population-based cancer registry and only one pathology lab services the entire country [30].

Haiti

Haiti is a conjoined Caribbean island shared with the Dominican Republic to its east, with a 95% African population of over 11 million inhabitants, including approximately 5.8 million women (~ 2million aged 30-79 years). Haiti is classified as a resource-poor, low-income country where most people live in poverty. In Haiti, BC presents at an earlier age and at an advanced stage, predicted anecdotally by DeGennaro et al. [32], and supported by their findings where more than half (53.8%) of the BC cases were in women aged 40-59 years at diagnosis (median 49 years), 49.2% premenopausal women, and 83.9% of cases diagnosed at stage III/IV. Fadelu et al. [33] also observed a median age of diagnosis at 49 years with 64.2% being premenopausal, as with Gomez et al. [57], where the median age at presentation was 48.5 years among their Haitian cohort in contrast to a median of 54 years in their Miami immigrant cohort. DeGennaro et al., (2018) [32] contend the unavailability of Haitian population data to be able to calculate age-adjusted rates for significant analysis. Body mass index (BMI) did not seem to have an impact on disease-free survival among a cohort of Haitian women with BC where 63% were either overweight or obese [58]. In the cohort studied by O'Neill et al. [59], 98% of persons were employed before illness, while 89% suffered a loss of income due to BC. O'Neill et al. [59] also found that 52% self-reported that they went into debt arising from their medical condition, out of a cohort of patients who were accessing free BC treatment at the time of the study.

Jamaica

Jamaica, with predominantly (90%) African ethnicity, comprises 14 parishes with a population of approximately 2.9 million inhabitants according to post-census estimates by the Statistical Institute of Jamaica [36]. It has one of the highest incidence rates for BC in the Caribbean, with a reported age-standardized rate of 43 per 100,000 in 2007 (the most current year available) [35]. The majority of the recorded cases were from Kingston and St. Andrew (57%). The annual increases in BC mortality rates among younger (35-44 years) and older (≥75 years) Jamaican women is alarming given that these age groups are typically excluded from the recommended guidelines (from 40 to 74 years) for BC screening used by healthcare providers in Jamaica [36]. Lerner-Ellis et al. [60] reported the mean age at diagnosis to be 49 years (range: 26-76) and 53.1% (94/177) premenopausal women diagnosed with BC.

Suriname

The Republic of Suriname, a former Dutch colony, is a Caribbean territory situated in the Northeastern part of South America, with an ethnically diverse population of around half a million people [43]. The highest incidence was seen in Creole Surinamese (35.7 per 100,000) followed by Javanese (Indonesian descent) (20.8) and Hindustani (Indian descent) (18.2) [43]. This study observed the highest incidence among women aged 40-49 years (n = 108), with 94 cases for the 50-59-year and 60-69-year age groups [43].

Trinidad and Tobago

With a population of approximately 1.4 million, of which nearly 690,000 are women, the twin-island state of Trinidad and Tobago is the second-largest English-speaking country in the Caribbean [61,62]. The ethnic composition of the population is estimated as follows: 34.2% of African descent, 35.4% of Indian descent, 23% of mixed ethnicity, and 8.4% of other ethnic backgrounds. A notable consideration that can potentially affect the dynamics of the BC population is the recent mass immigration of Venezuelan refugees to Trinidad and Tobago’s borders. It appears that women in Trinidad and Tobago are more likely to be diagnosed with BC at a younger age compared to American women based on findings from a diagnostic screening mammography study of over 2,500 Trinidadian women [63]. To further support this, over 80% of a study population of unselected BC patients were diagnosed between the premenopausal ages of 30-49 years (mean: ±43 years) [64].

While Pasquali [61] observed no racial differences concerning treatment or survival, a subsequent study using the same dataset found that ethnicity and spatial features seem to be strong predictors of incidence and mortality rates [48]. Women of African ancestry had almost twice the incidence and mortality rates (incidence rate: 66.96; mortality rate: 30.82) than women of East Indian (incidence rate: 41.04, mortality rate: 14.19) or mixed ancestry (incidence rate: 36.72, mortality rate: 13.80), with women residing in the North West Regional Health Authority catchment area followed by the North Central Regional Health Authority exhibiting the highest incidence and mortality rates for BC [46,48,49]. More than 10 years prior, a smaller study projected similar ethnic predispositions based on their findings; Afro-Caribbean women were the most affected group (54%), with Indo-Caribbean women accounting for 35% of cases along with mixed ancestry 11% (n = 299 women with breast carcinomas) [65]. Another interesting finding was that women of East Indian and mixed ancestry experienced significantly longer survival than those of African ancestry [48].

Summary

Overall, nine of the 31 Caribbean territories have published information on the demographics of BC patients. The demographics of interest reported were ethnicity, age of onset of BC, and socioeconomic status. The age of onset was the oldest in the Bahamas (56 ± 10 years), and predominantly among low-income communities compared to Trinidad and Tobago with a mean age of onset of 43 years; however, the socioeconomic influence was not determined. Otherwise throughout the Caribbean the reported age of onset was approximately 49 years and prevalent among premenopausal women. Ethnicity among the Caribbean territories was 80-95% of African ancestry, except for Trinidad and Tobago with 36% African ancestry. Clearly, more information on the age of onset and socioeconomic status among the ethnic groups are required to better understand the demographic etiology of BC.

Clinicopathology

Clinicopathological features of BC are critical to defining prognosis and guiding treatment. Earlier age of onset at advanced stages is typically associated with more aggressive forms of BC. Histological classification of tumors using immunohistochemistry provides information on the presence or absence of specific receptors, namely, estrogen (ER), progesterone (PR), and human epithelial growth factor receptor 2 (HER2), with an absence of all three is classified as one of the most aggressive BCs termed triple-negative breast cancer (TNBC). This helps to formulate the foundation for treatment.

Caribbean-born black immigrants diagnosed with BC living in the United States have an improved overall survival when compared with American-born black patients who had more ER-negative (ER-) BC (31.4% vs. 39.1%; p = 0.018) and TNBC (19.6% vs. 27.9%; p = 0.003) [66]. In contrast, Sung et al. [67], using U.S. population datasets found similar prevalence of ER- BCs between U.S.-born blacks and Caribbean-born blacks, with TNBC prevalence rate ratio (PRR) of 0.92 (95% CI: 0.81-1.04; p = 0.18) for U.S.-born blacks and PRR of 0.87 (95% CI: 0.78-0.98; p = 0.02) for Caribbean-born blacks with invasive BC [66,67]. Caribbean-born patients had more estrogen-positive (ER+) and progesterone-positive (PR+) BCs, presented at more advanced stages III/IV (44.2% vs. 35.2%; p = 0.016), and received more radiation and chemotherapy compared to the U.S.-born patient cohort, with no significant differences in inherited germline mutations among both groups in this study [66]. Furthermore, women born in the Caribbean had significantly worse survival in comparison to their counterparts born in the United States, independent of their ethnic background; a significantly lower BC survival was observed in African-Caribbean-born women living in the Caribbean (hazard ratio [HR]: 1.8; 95% CI: 1.6-2.1) versus African-Caribbean-born women living in the United States (HR: 1.3; 95% CI 1.1-1.7) versus African-descent women born and living in the United States, suggestive of biological, behavioral, environmental, and clinical risk factors affecting BC outcomes in women of Afro-Caribbean descent [68]. In Latin America and the Caribbean (LAC), 41% BC women (n = 221,255) from 22 countries diagnosed from 1966 to 2017 presented at stages III-IV, with a tendency of higher proportion in Caribbean cases than South American cases; the reason for this trend remains unclear [69]. The high percentage of advanced stage (III-IV) diagnosis in LAC contrasts sharply with the proportions observed among women from Western European countries [69].

The Bahamas

Research showed a positive correlation between age and tumor size, where 71.4% of all cases diagnosed with BC had a tumor size of ≥2 cm, with higher occurrence in the left breast than the right [13]. Ductal carcinoma (NOS and invasive) was the most common histological type observed, with most cancers occurring in grade II or higher (2009: 45.74%; 2010: 62.65%; 2011: 72.34%) in more than half of all diagnosed cases (elevated in 2011 to 83%) [13]. Late-stage (stage II) presentation was seen in 70% of cases, particularly in women aged ≤40 years (95.5%), while early-stage (≤stage I) diagnoses accounted for 16.7% of all cases [13].

Barbados

Invasive BC is also common among Barbadian women with one study reporting 9.8% in situ carcinomas and 90.2% invasive carcinomas [16]. Though sharing similar ancestral origins, variability between Barbadian-born BC patients and African American BC patients can be linked to varying exposures to certain risk factors. For example, later age at first birth, nulliparity, a history of benign breast disease, and a family history of BC are significant risk factors for the disease in this population [54]. Other reproductive variables such as age at menarche, lactation, and use of exogenous hormones were not significant predictors and may have influenced the lower incidence of BC in postmenopausal women [54]. A strong association between family history and BC suggests that genetics plays a significant role that supports the complex, multifactorial nature of BC, and is therefore likely the result of interacting genetic and environmental factors [54]. Tall stature increased risk among women aged ≥50 years (OR: 2.16, 95% CI: 1.02, 4.58), and a dual effect with age was suggested for both waist circumference and waist-to-hip ratio (decreased risk for those aged ≤50 years; increased risk among those aged ≥50 years) [70]. Interestingly, BMI did not influence risk in this population, where obesity is highly prevalent [70]. Hercules et al. [17] found that the mean age at diagnosis was 57.9 ± 14.2 years for non-TNBC, with 629 patients being diagnosed with grade 2 carcinomas and 608 being diagnosed with grade 3 carcinomas (47% and 45%, respectively, of the 1,342 patients with known grade information between 2007 and 2016). A high prevalence of TNBC (25% of cases with known receptor status) was noted, with a slightly earlier mean age at diagnosis of 55.5 ± 13.1 years compared to non-TNBC, and 72% TNBC was recorded as grade 3 carcinomas, also prevalent among HER2-enriched patients, whereas, for non-TNBC patients, only 39% of cases were grade 3 carcinomas (p < 0.0001) [17].

French Guiana

Most women (44%) presented with an intermediate prognosis, 39% diagnosed at stage 3 and 92% cases were invasive BC between 2003 and 2006 [29].

Haiti

Immunohistochemistry tests for receptor status (ER/HER2/PR) of tumors are unavailable in Haiti and are outsourced in the United States (reliant on patient affordability) making clinicopathologic data limited in both quantity and quality [32,71]. DeGennaro et al. [32] reported invasive ductal carcinoma in 87.3% of women, with a prevalence of 51.8% ER+ tumors (supposed overestimation due to over fixation), 19.6% HER2+ tumors, and 38.5% TNBC. The prevalence of 83.9% by late-stage diagnosis mentioned earlier (Patient Demographics) corresponds to Fadelu et al. [33] whose cohort had 53.4% late-stage tumors (given that 23.5% were of an unknown stage). In addition, George et al. [71] found the highest percentage of advanced-stage disease in Haiti in comparison to other Caribbean countries studied, where 64.7% (44 of 68) had stage III/IV BC. In 2010, Kobetz et al. [72] paralleled 45% regional/distant metastatic BC cases at diagnosis among Haitians living in Miami to only 10% among comparable ethnic/sociodemographic neighboring groups.

Jamaica

The first study in Jamaica to characterize clinicopathologic features associated with BC reported relatively young females presenting with a palpable lump, carrying a diagnosis of benign disease (70.4%) mostly attributable to fibroadenoma (39.4%) or nonproliferative disease (19.3%), along with a low prevalence of clinically significant premalignant disease, with invasive ductal carcinoma (69.5% cases) being the most common [73]. A later study by the same author described a remarkably high presentation of invasive tumors (93%) within the patient population (nine out of the ten males included), postulating that this could be the result of late-stage detection from palpable tumors as opposed to lesions detected by screening mammography [74]. These among other key researchers have asserted the need for a National Screening Program to reduce mortality and improve patient outcomes in the screened regions of the world [73,75]. A more recently published retrospective study found that a significant proportion of BC women present with large primary tumors with extensive nodal involvement, without distant metastases, to confirm suspected BC diagnosis among Jamaican women via detection of a breast lump (91% of cases) and invasive ductal carcinoma as the most common histologic diagnosis [76]. Most BCs were ER+ (62%) with one-third being ER+/HER2-, and most were diagnosed at stage II (57%) of the disease [76]. In 2017, 64.5% showed ER+ BCs, of which 24.3% were HER2+, while 26% were ER-/HER2- [60]. Conversely, in Jamaica-born immigrant women, ER- tumors showed prevalence similar to U.S.-born black women and West-Africa-born women with invasive BC, possibly due to a shared common ancestry (17-18th-century colonial slave trade) [77]. Two studies found the average tumor size to be 3.5-4.1 cm [74,76]. Delayed diagnosis is synonymous with poor prognosis; published five-year relative survival for BC forecasts worsening outcomes at later stages of the disease [76,78]. PR testing was unavailable in Jamaica during the period under study; therefore, the prevalence of TNBC could not be reported. However, efforts are being directed towards establishing the TNBC population in Jamaica [76,78].

Suriname

Invasive ductal carcinomas were more common (94%) than lobular tumors (1.7%), of which >60% presented at stage two or higher, the majority were grade 3, and 41.6% of patients had lymph node involvement at the time of diagnosis [43]. The hormone receptor status of most cases was unknown (350/421) because of the absence of immunohistochemistry technology in the pathology laboratory in Suriname [43].

Trinidad and Tobago

In 1985, 21% of the biopsies under investigation over a five-year period in Trinidad were malignant, with a high prevalence of fibroadenoma among benign breast lesions, more common among adolescents [79]. Following this, in the same year, a study of female breast biopsies under the age of 30 and another with persons between the ages of 12 and 20 also found that fibroadenoma occurs at an earlier age and is most common, followed by mammary dysplasia, whereas fibrocystic disease was found to increase with age, synchronous with clinical findings. Differentiation between benign lesions and carcinomas is clinically significant to avoid unnecessary mastectomy in adolescents and to guide critical treatment decisions [80,81]. This trend continues as 76% of benign breast diseases were fibroadenomas (34%), affecting mostly 15-20-year-olds [82]. Mammary dysplasia (42%) was most common in the 21-50 age group, with no significant differences by race [82]. The typical presentation is a painless lump [83] which could be attributed to most cases being self-detected among Trinidadian women.

From 1995 to 2005, more than half (1,328 cases) of diagnosed BC patients were between the ages of 40 and 59 years (13% <40 years), 83% were diagnosed at the localized stage (stage I to III), predominantly of the ductal phenotype [61]. Between 2002 and 2004, invasive ductal carcinoma was the most commonly occurring histologic type (70%; n = 299), with invasive lobular carcinoma accounting for 17%, which was unusually high compared to the global prevalence of invasive ductal carcinoma (≤4%) at the time [65]. Afro-Caribbean women were twice as likely to develop invasive ductal carcinoma and invasive lobular carcinoma compared to Indo-Caribbean women [65,83,84]. Consistent with global trends, subsequent studies over the years continue to realize dominant ductal carcinomas (NOS and infiltrating/invasive types) among BC cases [44,46,47,64,85]. Many BCs recorded in the national registry (n = 2,614) were aggressive; nearly 50% of cases were of regional or distant staging, with 54% being hormone receptor-negative, all of which contributed to poor survival outcomes regardless of treatment modality [61]. More Trinidadian women present with distant metastases (8.4%) compared with American women (5.0%) [86].

In Jamaica, a small study reported a significant prevalence of human T-cell leukemia virus type 1 (HTLV-1) seropositivity among persons with BC; however, a comparative study in Trinidad and Tobago found no association of the retrovirus HTLV-1 with BC patients compared to the general population [85].

Summary

Overall, seven out of the 31 Caribbean territories published papers on the clinicopathology of BC in the respective sample populations. Of interest were the type of carcinoma, size, stage of diagnosis, presence of hormone receptors, and TNBC. All seven countries showed invasive ductal carcinoma as the predominant diagnosis. The majority of patients in Haiti, French Guiana, and Suriname were diagnosed at stage 3 compared to stage 2 for Barbados, The Bahamas, and Jamaica. The carcinomas found in Jamaica were 3.5 to 4.1 cm in size, nearly double that of The Bahamas with a mean size of 2 cm. TNBC was only reported for Barbados and Haiti. The Bajan BC samples showed a higher incidence of TNBC (45% to 47%) compared to Haiti (38.5%). The Jamaican and Haitian BC samples showed ER+ receptors for 62% and 52%, respectively. Interestingly, all hormone receptors were absent in 54% of the Trinidad and Tobago BC samples, which is indicative of the presence of TNBC. Studies on establishing population-specific routine clinicopathologic methods can assist in understanding and treating BC in the Caribbean. Variability in BC profiling among nations, particularly in countries where immunohistochemistry is unavailable, lends support to the urgent need for standard protocols in BC management for the Caribbean, perhaps better achieved through a unified effort.

Genetics

BC predisposition gene testing allows the identification of individuals at high risk for BC who may benefit from increased surveillance, chemoprevention, and prophylactic surgery. A review of the existing literature on BC genetics for LAC established the need for economically feasible population-mutation-specific panel testing where large genomic rearrangements are included and proper clinical infrastructure including genetic counseling is widely available to benefit from genetic-based BC prevention strategies [87]. The Caribbean Women’s Cancer Study is a cross-sectional study of patients with invasive BC and/or ovarian cancer born in the Caribbean. Their most recent publication based on a multisite genetic association study discovered a one in seven chance of having hereditary breast and ovarian cancer among Caribbean-born individuals with breast and ovarian cancer [71]. This supports the recommendation for multigene (panel) testing in this region, particularly for individuals with a family history of BC.

The Bahamas

A high prevalence (24% to 27%) of *BRCA1* and *BRCA2* mutations is estimated among all Bahamian women with BC, the highest reported mutation prevalence for any country studied to date [53]. An earlier study for the same sample population reported a steep 45.3% *BRCA1* mutation frequency in women diagnosed before the age of 50 years, 41% in women with a first-degree relative with breast or ovarian cancer and 58% in women with bilateral BC, concluding the need for genetic testing among BC patients in The Bahamas [88]. This study discovered six African founder *BRCA1* mutations (IVS13+1G>A, 943ins10, IVS16+6T>C, and 5443T>G) [88]. A seventh recurrent, novel *BRCA2* mutation (8128delA) was also found in the follow-up study, which increased the prevalence of founder mutations in the Bahamian population from 22.9% to 25% of unselected BC patients [53]. A total of 58 mutations (49 among unrelated women) were discovered with double the mutation prevalence (52%) in women diagnosed at age ≤39 years than in women diagnosed from age 40 to 49 years (26%), where 128 out of 214 women involved in the study had a family history of BC [53]. To further explore this, the study was expanded into the healthy women community (n = 1,847), which resulted in a 2.8% *BRCA1/2* mutation prevalence among unaffected women with a first- or second-degree relative with breast or ovarian cancer in The Bahamas [89]. Additionally, a truncating *RAD51C* mutation in five cases (1.3%) and no truncating mutation in controls (5/387 versus 0/653; p = 0.007) was detected, which continues to uncover the relatively high genetic predisposition to BC in this population [90]. None of the *RAD51C* mutation-positive BC patients declared a family history of ovarian cancer (known association) whereas two patients reported multiple affected first- and second-degree relatives with BC (unclear association) [90].

Barbados

Zhang et al. [91] found no *BRCA1* mutation carriers among a cohort of 118 Barbadian BC patients who were genotyped for six of the recurrent *BRCA1* discovered previously among Nigerian patients with invasive BC in a study conducted in 2012 to identify recurrent *BRCA1* and *BRCA2* mutations in BC patients of African ancestry.

Cuba

A 2008 study identiﬁed a deleterious *BRCA1* or *BRCA2* mutation in 2.6% (1 in 40) of unselected Cuban women with BC (n = 307) and reported four common mutations: *BRCA1 185delAG*, *5382insC*, and the *BRCA2 6174delT* mutations common to Ashkenazi Jews and others of eastern European ancestry [21]. Overall, 87.5% of mutation-carriers had either early-onset BC (diagnosed at age ≤50) or had a ﬁrst- or second-degree relative with BC [21], supporting the hereditary impact and the need for genetic testing. A researcher with a focus on community genomics, specifically BC genetics, has demonstrated a grave lack of knowledge about “genes” and “genetics” with fewer BRCA mutations among Cuban women, pinpointing a major part of the challenge in cancer diagnostics and personalized medicine [92,93].

Dominica

In Dominica, George et al. (2021) [71] reported 4 of 61 BC patients had a germline variant in *BRCA2* and *PALB2* recurrent variants.

Haiti

George et al. (2021) [71] found 6.7% (5 of 75) of Haitian BC patients had a variant in *BRCA1*, *BRCA2*, or *PALB2* with the second-highest distribution of *BRCA2 *germline mutations.

Jamaica

The most recent genetic study conducted in this population revealed lower than expected *BRCA1/2* (1.7%) mutations, especially given the predominantly African ethnicity, whereas the highest reported to date for *PALB2* (2.8%) among unselected BC patients (n = 179) [60]. New *PALB2* mutations (c.109C>A, c.43G>T, and c.2502delC) were discovered by this research, and no common mutations were seen [60]. Familial history discovered 82 of 175 (46.9%) patients had a female relative with BC, whereas 12 of 177 (6.8%) patients had a female relative with ovarian cancer [60].

Trinidad and Tobago

The first survey of hereditary BC in Trinidad and Tobago reported 28 of 268 (10.4%) mutation-positive invasive ductal carcinoma patients as follows: 15 (53.6%) *BRCA1* mutations, 10 (35.7%) *BRCA2* mutations, two (7.1%) *PALB2* mutations, and one case with both *BRCA2 *and *PALB2 *mutations; overall, there were 29 mutations, four of which were seen twice in unrelated patients [64]. A follow-up of this study tested 76 consenting at-risk relatives (ARR) of the 24 mutation-positive carriers (probands) and showed that 35 (46%) tested positive for family mutation [94].

Summary

Overall, seven out of the 31 territories had published genetic tests done for BC patients, all of which were conducted by the same research group. The markers *BRCA1*, *BRCA2*, and *PALB2 *were identified from the literature. The Bahamas had a much higher prevalence of *BRCA1* and *BRCA2* (24% and 27%, respectively) compared with Cuba, Dominica, Haiti, Jamaica, and Trinidad and Tobago (0% to 6.7%). Interestingly, none of the BC patients in Barbados tested positive for *BRCA1*. Although the ratio of *BRCA1/2* was similar between Jamaica (1.7) and Trinidad and Tobago (1.5), the presence of *PALB2 *was much higher in the Jamaican sample (2.8%) compared to the Trinbagonian sample (0.75%). The genetic information on BC in the Caribbean needs further investigation with larger sample sizes and familial cohort testing. This will help toward the understanding of the inheritance pattern of BC markers and to assess environmental factors influencing disease presentation.

Behavioral risks

Over a decade ago, Caribbean-born women were much less knowledgeable about BC compared to American and Eastern European women holding such unacquainted beliefs as BC is always fatal and surgery causes cancer to spread [95]. Currently, adherence to recommended mammographic screening remains least compliant among Caribbean-born women than their counterparts in other regions of the world; demographic, healthcare system, and psychosocial variables help explain these disparities [96]. Barriers to timely BC screening in the Caribbean have been attributed to determinants of health such as lower levels of education and less access to healthcare, which likely influences BC morbidity and mortality in the region [97]. Some studies demonstrate that individuals with existing comorbidities, such as diabetes, have lower cancer screening rates than those without comorbidities, supported in the Caribbean, with only 50.8% hypertensive and 22.3% diabetic patients among the eligible group (52%, n = 841) who reported timely screening mammography [97]. George et al. [71] reported a higher rate of self-detection (86%) of breast masses over screening mammograms among Caribbean-born women.

In the Caribbean, alcohol intake and higher BMI were found to be more strongly associated with lower socioeconomic status, and unmarried women had a higher tendency to be diagnosed with BC over married women [8]. The impact of alcohol consumption, a modifiable BC risk factor, also had a confirmed positive relationship with mammographic density [98]. Quandt et al. [98] observed that high levels of alcohol consumption (more than seven servings/week) were associated with an increase in percentage density including fully adjusted confounders models (though not statistically significant), but no association was seen between alcohol consumption and nondense areas [98]. There were no observed differences by race/ethnicity for percentage density in their cohort that included Afro- and Hispanic-Caribbean women living in the United States; nevertheless, this study had a small sample population (n = 189) with narrow ethnic diversity and was limited in the number of women who reported consuming more than seven servings/week (n = 14), which contributed to large CIs [98].

Barbados

One report screening 6,207 women aged ≥60 years from the large SABE study found a two-year mammography rate for Bridgetown of only 18.5% (n = 993) [99]. In the absence of a national screening program, limited public awareness and knowledge force Barbadian women to actively seek information about BC and the screening process from other available public health information on diabetes and HIV/AIDS to gain understanding about mammography, even though subsidized mammography is available within the public sector [100]. This level of incognizance is outrageous considering the highest BC incidence and mortality rates in the region. Women believe that health professionals’ attitudes have a significant impact on their health-seeking behavior as some who had experienced a mammogram described frustration with medical professionals (varied free-public vs. fee-private) and had not received enough information to make an informed decision [100]. Other barriers to uptake include fear of consequences of the pain accompanying the actual mammographic procedure and the subsequent BC diagnosis linked to societal taboo, public stigma, and negativity/ostracism (on account of public ignorance about BC) [100].

Cuba

Reyes-Ortiz et al. [99] reported that prevalence of mammography use in older women (≥60 years) was only 9.8% in Havana (n = 1143). However, Galan et al. [101] confirmed that cancer incidence and mortality (including BC) remain high in Cuba, even though a screening program with high coverage has been in place since 1968.

French West Indies

A 2014 cross-sectional national survey reported a high rate of BC screening (81.5%) in the French West Indies parallel to mainland France; an organized screening program is available in Martinique (since 1991) but not in Guadeloupe [102]. Higher BC screening participation was reported among women having hot water at home and having visited the general physician in the last year [102]. In a study to better understand the knowledge of BC patients regarding BC risk factors, most treated BC survivors did not have any opinions about the causes or risk factors of BC, but those who had an opinion reported stress, genetic factors, radiation, and pollution as the most important causes [24].

Grenada

A qualitative cross-sectional study found that groups of women in Grenada with varying belief patterns and sociodemographic characteristics shared different viewpoints, specifically higher versus lower uptake of breast self-examinations [56]. Younger women and those who did not attend church were motivated to perform breast self-examinations, whereas frequent churchgoers and divorced/separated/widowed women felt more susceptible to BC, were less confident, and saw less benefit in self-examinations [56].

Haiti

DeGennaro et al. [32] noticed that over half of the women in their program living in Haiti did not seek medical intervention until >12 months after first noticing a breast lump. Numerous studies conducted as early as the 1980s revealed extremely low compliance to mammography screening among immigrant Haitian women (many having never been screened), despite improvements in cancer screening in the United States, owing to severe lack of awareness about BC screening, psychosocial factors such as fatalism, and misguided beliefs about BC, as well as demographic and healthcare constraints, in addition to immigrant-specific barriers such as language, documentation status, and insurance eligibility [72,95,96,103]. Gany et al. [104] surveyed providers serving the Haitian immigrant population in New York City and reported gaps in the consistency of clinical practice concerning BC examinations and screening referrals. Interestingly, Kobetz et al. [72], in their survey of Haitian women living in Little Haiti, Miami, discovered that 42% (396/940) of participants had a mammogram in the last two years, a higher-than-expected finding for this population.

Jamaica

With a notably high BC mortality rate among Jamaican women, remarkably, there is no national breast screening program, though screening mammography has been shown to reduce these deaths by as much as 50% [35,36,105]. Low patient and physician participation in screening mammography may also be contributory [75]. Although Jamaica adheres to the American Cancer Society guidelines of beginning screening at 40 years of age, women between the ages of 25 and 59 years formed the largest BC population from 1998 to 2007, with startling mortality rates observed among the 35-to-44-year age group in 2014 [34-36,75].

In 2006, 88 of the 274 women (32%) interviewed admitted to being screened via mammography for the first time, whereas the majority had repeat mammograms, often delayed for over a year because of perceiving the need for a doctor’s referral (p < 0.001) [106]. A mere 5.8% of 121 BC patients reviewed during 2006-2007 at the University Hospital of the West Indies conveyed a history of previous screening mammograms [78]. Overall factors influencing improved mammographic experience were pain intensity (odds ratio [OR] = 0.84; p < 0.04), interval status of previous mammography (OR = 2.24; p = 0.059) and knowing someone with BC (OR = 0.35; p < 0.04); conversely, fear, pain during mammography, subjective indifference, inertia, and reliance on physician referrals were identified as barriers to compliance with mammographic screening guidelines [106]. Despite these, the lack of screening is related to relatively high costs as mammography screens are only available privately [107].

Trinidad and Tobago

In Trinidad and Tobago, there is no population-based screening program; however, self-examination and clinical recommendations have improved over the years. A study in 1996 conducted in the chiefly African-populated island of Tobago (the smaller island) where no mammography services were available at the time found low levels of self as well as clinical breast examinations largely due to the unavailability of programs for early detection (66% of respondents), cost of screening (52%), no direct access to mammograms with the hassle of traveling to Trinidad for screening (66%), and fear of cancer (48%) [108]. Trinidadians are now more aware of the need for regular screening and over 90% are willing to have repeat mammograms, though less than 50% knew that mammograms are not always capable of detecting cancer [86,109]. A study in 2014 conducted in South Trinidad, with a more diverse study population, found much higher compliance with routine self-breast examinations, and education and family history of breast disease were strongly associated with a better attitude towards mammography usage [86]. Self-examination has been proven to be essential with an alarming 91.6% (245/268) of BC cases in a Trinidad and Tobago study population detected via this routine procedure [64]. However, Trinidadians reported that fear of finding cancer discouraged screening [86,108,109].

Summary

Overall, seven out of the 31 Caribbean territories had some coverage on behavioral attitudes towards BC. Jamaica and Haiti notably did not have an active national screening program. In addition, both Jamaicans and Haitians on discovering a lump would delay mammograms up to more than a year. Interestingly, the study from the French West Indies and Grenada showed that access to hot water and being a part of a church, respectively, greatly improved self-examination and detection. In Trinidad and Tobago, self-examination was mostly responsible for BC detection. This section clearly shows the dearth of attitudes, approaches, and knowledge at the national, clinician, technician, and patient level that need to be addressed to improve detection and early treatment of BC in the Caribbean.

Diagnosis and treatment

Numerous studies have charged the Caribbean region with derisory early-detection measures and a dearth of national BC screening programs to mitigate the high mortality of this disease. Early detection of BC is well known to increase chances of survival and is therefore reliant on national policy and intervention for management. A survey conducted in 2006 by the Latin American and Caribbean Society of Medical Oncology in 12 countries with BC specialists reported a delay over three months between the suspicion of cancer and a mammogram or clinical examination in the region, not to mention the longer times between diagnosis and treatment initiation, all of which could account for the higher percentage of late-stage diagnosis and poor survival in this population [69]. There are no medical oncologists practicing in seven of the countries, and only ten countries in this region have radiation oncologists [9]. Following a lumpectomy for invasive BC, evidence indicates local recurrence in 30% of patients who do not receive radiotherapy [110]. This is challenging for many Caribbean nations with no available radiotherapy (17 out of 31 islands) [9], while access to existing facilities is inadequate [110]. Local recurrence was highly prevalent in a study population of Caribbean immigrants treated at a breast clinic in the United States due to the lack of radiation therapy in conjunction with lumpectomies and mastectomies performed in the country of origin [110]. Neutropenia, a common complication of chemotherapy characterized by low levels of neutrophils, is prevalent in African and Afro-Caribbean populations [111]. Grann et al. [111] found the prevalence of c199a neutrophil elastase polymorphism (associated with severe congenital neutropenia) ranged from 2.5% among American-born white women to 54.8% among women from Barbados or Trinidad and Tobago (p < 0.0001). This study suggested ancestral and genetic linkages to neutropenia, vital to guide the treatment of BC patients.

The WHO estimates 78% of 40 million people who need palliative care live in low- and middle-income (LMIC) countries, which includes 30% to 50% of patients presenting with advanced-stage BC diagnosis (III-IV) in LAC [112]. High mortality is largely due to insufficient access to treatment and patients presenting with incurable advanced stages of the disease [112], as presented by this review. A BC diagnosis can be associated with deficits in social, psychological, financial, career, and spiritual well-being, thus palliative care has been widely acknowledged as a means to offer holistic management and relief of such serious health-related suffering among patients with serious illnesses, typically cancer, as well as attending to the patient, his or her family, and end-of-life care [112,113]. The Breast Health Global Initiative, the National Comprehensive Cancer Network, and the American Society of Clinical Oncology have proposed the integration of palliative care services that are aligned with local needs and resources to provide a strategy that is culturally and resource appropriate (resource-stratified) through provider education and training [112].

The Bahamas

Among Bahamian BC patients, surgery was the preferred method of treatment (later confirmed by a report of 79% (110/194) of BC participants who had a surgical mastectomy), with modified radical mastectomy being the top choice, in contrast to radiation used only in 6.5% of patients, with no patient on record for radiotherapy in 2011 [13,113]. A follow-up of *BRCA1/2* mutation carriers revealed a lower than preferred (three out of 13) uptake of preventive surgery among unaffected persons and 46% (36 out of 78) overall which shows promise regarding the effectiveness of the genetic program [114].

French Guiana

BC patients typically require specialized care that is covered outside of French Guiana in either France or Martinique (other French territories). Roue et al. [29] observed that 96% of women benefitted from treatment within eight weeks of diagnosis in the form of surgery (84% curative), followed by chemotherapy (54%), and to much lesser extent radiotherapy (8%) and/or hormone therapy (9%).

Haiti

Based on the literature, BC diagnosis and treatment in Haiti is extremely poor resulting in advanced stage at diagnosis and limited clinicopathologic classification. Haitian cancer patients suffer gravely from limited access to healthcare, largely due to high costs of treatment, specifically chemotherapy drugs, and no available radiotherapy services [115]. O’Neill et al. [59] showed that even when healthcare is offered for “free” to BC patients, the nonmedical expenses (transportation, lodging, etc.) to access care can be deemed catastrophic (to quote their term). In recognition of the demand for BC care in Haiti, Project Medishare (nonprofit) launched a BC treatment program in Port-au-Prince in July 2013 [115]. This treatment program aims to improve BC healthcare through crucial partnerships aimed at strengthening and equipping the local medical community. DeGennaro et al. [115] suggested plans to establish a local pathology laboratory to meet the need for immunohistological classification of tumors and a radiotherapy treatment center, both of which were unavailable in Haiti at the time of this study. Fadelu et al. [33] established the continued absence of radiation therapy and therefore unavailability of breast-conserving surgery, as well as the absence of HER2-directed therapy and targeted therapies [57]. DeGennaro et al. [32] later described a high level of recurrence among Haitian BC patients after treatment (primarily having a mastectomy only) who presented to them for adjuvant chemotherapy and/or hormone therapy. Patients with stage IV disease had 81.5% 12-month survival rate and 53.6% 24-month survival rate [32]. Gomez et al. [57] observed that women with BC living in Haiti had significantly worse outcomes than Haitian immigrants living in Miami, seemingly related to more advanced stage at presentation, younger age at diagnosis, high prevalence of ER-negative tumors, and lack of timely effective treatments. Though identifying Haiti as a resource-poor, low-income county with inadequate healthcare, Fadelu et al. [33] advocated the possibility of curative treatment for BC patients.

Jamaica

During 2006-2007, 65% of BC patients were referred to the University Hospital of the West Indies for assessment for adjuvant therapy after BC surgery, 20% for preoperative systemic therapy (in locally advanced breast cancer [LABC] cases), and 15% for management of the metastatic disease [78]. The most utilized neoadjuvant chemotherapy regimen was adriamycin/cyclophosphamide with a sequential taxane (paclitaxel) (59%); 41% of patients did not receive a taxane, primarily due to cost-prohibition, at the time of this study [78]. All ER+ BC patients were prescribed endocrine therapy upon completion of chemotherapy, and two HER2+ patients were prescribed adjuvant trastuzumab but were unable to complete the recommended course due to financial restrictions [78]. Further, 47% of patients suffered recurrence within eight to 18 months after completing preoperative chemotherapy [78]. Jamaica’s National Health Fund now provides subsidies for chemotherapy for BC, which has assisted the availability of adjuvant therapy and critically improved care for patients [78]. Given the relatively higher proportion of newly diagnosed LABC patients and the median tumor size reported from these studies [74,76,78], there is a need to implement BC policies on the management of LABC, so that eligible patients may be considered for neoadjuvant systemic therapy in adherence to international recommendations [78]. Gram-negative organisms were noted to be the predominant isolates in febrile neutropenic episodes (common to developing countries compared to developed countries where gram-positive organisms prevail) in Jamaican cancer patients where there was no significant susceptibility by gender or cancer type [116].

Alternatively, a 2011 survey reported 73% use of medicinal plants to treat illness among Jamaicans, in alignment with the WHO’s 80% estimation in developing countries, as well as findings from a study conducted in 2016 where 80% (including 33/39 BC patients) of the interviewed population (n = 100) engaged in this practice [117]. The common practice of medicinal herbs in this region is largely due to tradition and availability as well as the lack of side effects compared to prescription drugs [117].

Palliative care, though currently limited in Jamaica, as in most other Caribbean islands (LMIC), has been significantly adopted into policy and is being delivered through small community-based private initiatives to manage BC burden [112].

Suriname

Surgery was the primary form of treatment for invasive BC, 87% mastectomy and 13% lumpectomy, according to van Leeuwaarde et al. [43], combined with adjuvant chemotherapy in 20% of cases and radiotherapy in 14% of cases. The introduction of national guidelines (absent during the study period) in 2005 to qualify persons for chemotherapy would increase this observed number and possibly lead to better outcomes than are currently experienced [43]. On the plus side, the median wait period for surgery following diagnosis is 17 days in Suriname, shorter than that in The Netherlands where the median wait period is almost a month [43]. However, there are long delays following surgery for adjuvant chemotherapy (nine vs. four weeks) and radiotherapy (11 vs. eight weeks maximum, four to six weeks preferable); these could be due to the shortage of cytotoxic drugs, delays by physicians/patients, and absence of radiotherapy facilities within the country [43], as experienced in Haiti, another low-resource Caribbean territory. Islam et al. [43] believe that the introduction of the Surinam BC treatment guidelines, the planned introduction of immunohistochemistry, and the anticipated introduction of radiotherapy in late 2010 are expected to enhance the diagnosis and treatment of BC.

Trinidad and Tobago

Most BC cases are primarily discovered through self-detection with the presentation of palpable breast lump(s) before confirmed clinical diagnosis. Over the years, mammography usage has increased but has not shown any significant contribution to the mitigation of mortality associated with BC inferred by the unreliability in differentiating benign tumors (accompanied by mastalgia) with malignant tumors [45].

From 1995 to 2005, through 2009, 95% (n = 2,614) of women received some type of therapy: 23% surgery only, 40% combination of two therapies, and nearly a quarter receiving all therapies (surgery, radiotherapy, and chemotherapy) [61]. Surgical procedures tend to be the most common approach used for BC management; patients who had surgery also had chemotherapy, radiotherapy, and hormone therapy in various combinations, with the combination of surgery and chemotherapy being the most common (n = 151; 23.6%) [47]. The most frequent surgical procedure was unilateral mastectomy (n = 321) which, in some cases, was combined with axillary node clearance (ANC) [47]. The introduction of a breast clinic in 2012 at a major teaching hospital in south Trinidad significantly reduced the mastectomy and ANC rate, often associated with significant morbidity and poor cosmetic outcome with both negatively impacting the quality of life of patients [44]. Breast conservation surgery (BCS) has been established as a safe option for most women with early BC, given that the five-year survival of BCS with radiation is not statistically different compared with mastectomy alone in patients with stage I or II BC [44]. Early hospital discharge following BC surgery is now a feasible option for most BC patients and can be safely implemented in a resource-limited setting, such as Trinidad and Tobago, where cost containment and public healthcare management are essential [45,118].

Cancer patients interviewed at two clinical sites in Trinidad who were treated with chemotherapy, surgery, and/or radiotherapy also reported use of a wide range of herbal remedies and functional foods for cancer treatment, health maintenance, and to counteract the side effects of conventional treatment, with the highest usage observed among persons with BC [119]. This study identified 55 herbal remedies, supplements, and functional foods, chiefly soursop (*Annona muricata* L.) (80.9%) along with wheatgrass (*Triticum aestivum* L.), saffron (*Crocus sativus* L.), Aloe vera (L.) Burm f., garlic (*Allium sativum* L.), and ginger (*Zingiber officinale* Roscoe), which are among the most commonly used by the sample population (n = 150 cancer patients) [119].

Summary

In total, six out of the 31 Caribbean territories had published information on the treatment and control of BC. Treatments included surgery, radiation, chemotherapy, hormone therapy, medicinal plants, and a combination of the five treatments. Surgery was by far the main treatment for BC in all six countries. Five countries, except Haiti, utilized some form of radiation, chemotherapy, or hormone treatment together with surgery or in place of surgery. These approaches may be a result of the stage at diagnosis and not necessarily the availability of treatment options. Publications from Haiti showed the expensive cost of radiation and chemotherapy medication/drugs as a hindrance in the treatment of BC. Jamaica and Trinidad and Tobago showed the patient-driven use of medicinal plants for the treatment of BC. Further work in the area of available treatment options and cost to the patient/system in determining treatment options needs to be done at a regional level. This would gear BC treatment towards improved patient quality of life and survivorship outcomes.

Breast cancer control

Although BC screening is widely available in the CARICOM Caribbean, services are not part of structured or comprehensive screening programs or follow defined national policies, as recommended by the WHO [6]. Spence et al. [9] reported that only seven CARICOM countries (Belize, Jamaica, Guadeloupe, Martinique, Puerto Rico, Suriname, and Trinidad and Tobago) have operational, stand-alone national cancer control plans. The Caribbean requires the collaborative support of the scientific and governmental bodies to urgently mount a communal proactive response to improve cancer surveillance and research with consideration to the unique needs of each Caribbean country, as they have varied urgency and capacity to respond to increasing regional rates of cancer [10] This need is emphasized in one study by the sparse and incomplete data, aging populations, high cancer risk prevalence, low/limited-resource settings, as well as high frequencies of cancer-related deaths or cancers with poor prognoses, or more so, the lack of targeted cancer care and prevention strategies, all of which highlight the importance for progress in cancer surveillance and research [10]. Spence et al. [9] corroborated that providing comprehensive and locally responsive cancer care is particularly challenging in the Caribbean because of the geographical spread of the islands, the frequently under-resourced healthcare systems, and the absence of a cohesive approach to cancer control, warranting a need for such an initiative.

Additionally, the widespread consensus from reports has found that Caribbean women are less likely to adhere to recommended BC screening, whether self-examination or mammography, crucial to effectively control BC. However, findings from the CanIMPACT study support other literature that women from LAC tend to be diagnosed with BC at a later stage and are less likely to have screen-detected cancer [120]. Consequently, unaccompanied screening is not adequate. Comprehensive BC control involves sufficient financial and human resources, infrastructure, and strong administrative structures that ensure quality services for the continuum of BC care and prevention that incorporates screening, early detection, diagnosis, treatment, and palliative care [15]. A usual source of healthcare and a woman’s island of residence were significantly associated with timely screening mammography (p < 0.05) [97]. For the defined Caribbean region discussed, individual countries have invested between 4.9% and 9.4% of their GDP in healthcare [15]. Palliative care education initiatives in partnership with well-established academic programs in successfully developed regions can vastly improve disease outcomes through resource-stratified palliative care training for Caribbean healthcare providers [112].

The Bahamas

A national genetic screening program (*BRCA1/2* variant founder-mutation panel) is being offered to manage the burden of BC among Bahamian women resulting from several studies that have discovered a high prevalence of *BRCA1/2* founder mutations among unselected affected BC persons in addition to unaffected women with a family history of breast and ovarian cancer [53,60,88,89]. However, it is expected that inexpensive next-generation sequencing will be included in this program to offer universal genetic testing for *BRCA1* and *BRCA2* [114]. As a measure to improve disease outcome, prophylactic treatment (such as salpingo-oophorectomy and bilateral mastectomy or hormone therapy) is offered to all mutation carriers in the Bahamas, on account of 80-90% lifetime risk of developing BC [13,114,121]. Responsiveness to genetic testing by ARR was more successful when contacted directly by a genetic counselor than through other means, which is not the standard practice in the United States and thus shows the need for culturally tailored initiatives [121].

Cuba

The National Program for Early Detection of Breast Cancer began in 1987 in Cuba. At the time, researchers fortified the need for all women over the age of 40 to be screened for BC, finding the purported risk factors to be ineffectually statistically significant, though acknowledging the resource limitations to national mass screening [122]. Cuba is known for having an evenly distributed comprehensive national healthcare system and for emphasizing preventive medicine with extraordinary compliance to cancer screening among Cuban women [19,21,122]. All cancer services are provided through national cancer treatment centers, and access to care is evenly distributed, standardized, and universal [21].

French Guiana

Mass BC screening was initiated in 2005 by the Guianese Association for Organized Cancer Screening targeting women from an earlier age than recommended in France (50-74 years) as >50% incidence is seen among women aged <50 years [29].

Haiti

The Innovating Health International Women’s Cancer Center in Port-au-Prince has partnered with Haiti’s Ministry of Health and local nonprofit organizations to launch an awareness campaign for self-breast examinations via grassroots community meetings, the Internet, and radio, including launching a Creole-language website (www.kanseayiti.com) in efforts to increase awareness and promote early presentation [32]. Tillyard et al. [123] showed the effectiveness of such avenues in reaching the community with results from their mixed-method survey as participants (both males and females aged 13-65 years) shared their means of accessing healthcare information through radio (63.5%), community health workers (62.3%), TV (46.6%), church (31.4%), schools (28.9%), family (20.6%), and the market (6.1%). Clinical and research programs are supported through a collaboration of the U.S.-based nonprofit and the University of Florida College of Medicine, Department of Medicine [32]. Meade et al. [124], in recognition of the importance of promoting culturally appropriate education and awareness of breast health among Haitians, who reside in a resource-limited, low-income country, used the CLEAN (Culture, Literacy, Education, Assessment, and Networking) systematic model to guide community outreach efforts that were considered successful in reaching thousands of women. Large differences in self-reported knowledge (low overall) of female cancers were discovered between the demographics by [123], which shows the need for such educational initiatives as well as supports the necessity for executing a guided culture-specific approach. This study also noticed that wealthier persons had more overall knowledge of BC than the most educated Haitians [123].

Jamaica

A digital theory-based educational intervention proved influential in improving behavioral risks associated with BC in Jamaica. The study reported statistically significant increases in knowledge of BC risk factors, symptoms, and types of screening, as well as screening rates in screening-naive Jamaican women [107]. Remarkably, there is less than 5% diagnosis of patients with LABC in regions with regular screening mammography compared to 33% LABC observed in Jamaican women [78].

Saint Vincent and Grenadines

Though no studies were found on BC control in St. Vincent and the Grenadines, two studies conducted by the same team among primary healthcare nurses found that their knowledge of BC risk factors, signs and symptoms, and screening recommendations were poor [40,41]. In the follow-up report, marked variation in BC screening practices was observed among the cohort of nurses surveyed [41]. This alludes to the situation concerning BC care in this country.

Trinidad and Tobago

An investigation conducted to determine BC diagnosis from screening in Trinidad and Tobago found a 5% BC prevalence among women who received digital mammography from the Trinidad and Tobago Cancer Society (TTCS) between 2009 and 2011 [63]. Most positive cases (63%) were between the ages of 40 and 59 years (36%, 40-49 years) [63], providing evidential support for beginning early-detection screening by age 40. Multivariate analysis among unaffected screened women associated BC diagnosis with a positive family history of BC, presence of symptoms, previous breast surgery, and increased breast density [63]. Data collected from the national cancer registry revealed an increase across all demographic groups, implying a need to improve cancer prevention, screening, and treatment options, as well as genetics and genomics-based research [48]. To address this major public health challenge, governmental efforts, such as the National Oncology Programme and nongovernmental bodies like the TTCS, have developed initiatives to increase screening for early diagnosis, awareness, education, and treatment [61]. Nevertheless, several limitations, not restricted to diagnostic delays/challenges, inadequate definitive care largely as a result of a shortage of technical persons, as well as other significant challenges across the cancer care continuum, render current early-detection BC screening unfeasible to the public in Trinidad and Tobago [45,47,49]. Confoundingly, only one out of 1,176 BC cases was diagnosed by mammography in the five-year period of 1995-l 999, as recorded by the national cancer registry [45].

Summary

In total, six of the 31 Caribbean territories showed published work on systems in place for BC control. Out of the seven countries, The Bahamas, Cuba, and French Guiana had national screening programmes for BC. Cuba had one of the oldest systems with equitable access and treatment options for BC, while The Bahamas utilized genetic screening and prophylactic treatment options for high-risk persons. Jamaica, Haiti, and Trinidad and Tobago relied on breast examination as the main form of control. Studies from St. Vincent and the Grenadines showed a lack of any type of screening program for BC control. BC control is important for early detection, treatment, and education of the Caribbean population. BC control systems need to be clearly documented, and adherence to these guidelines should be measured regularly.

## Conclusions

Although our review uncovered major variability in the incidence, management, etiology (e.g., the prevalence of genetic mutations), and mortality from BC among Caribbean countries, more rigorous studies focusing on prevention strategies and standardization of treatment approaches are required to guide enhanced surveillance with the expected improvement in patient outcomes. Low-resource Caribbean countries are burdened by more advanced disease with expected poorer BC outcomes (i.e., shorter periods of disease-free survival). Countries with established national cancer registries seem to have a better approach to the management of BC. The introduction of cancer treatment programs in association with international nonprofit groups has shown tremendous improvement in quality, accessible cancer care for nationals, particularly in LMIC settings.

Several studies have recommended the need for the implementation of national screening programs combined with education initiatives to promote BC awareness as early disease detection has contributed to consistent reports of decreased mortality rates in screened populations where incidence is higher than in the Caribbean. Comprehensive BC control involves prevention, early detection, diagnosis and treatment, rehabilitation, and palliative care. Palliative care education initiatives in partnership with well-established academic programs in developed regions may improve disease outcomes through resource-stratified palliative care training for Caribbean healthcare providers. Variability in the standard of care and lack of rigorous standardized monitoring and reporting of outcomes through state-of-the-art cancer registries are required to accurately reflect the true status of breast cancer in the Caribbean.

This study provides essential foundational support to researchers, healthcare planners, and administrators who are interested in the status of the Caribbean BC population of diverse ethnicities, environmental influence, immigrants, socioeconomic status, and sociocultural practices. It also allows an opportunity for epidemiological investigations that can provide deeper insights into the specific themes addressed in this review.

## Appendices

For the purpose of this review, the following 31 Caribbean Community (CARICOM) countries form the defined Caribbean region: Anguilla, Antigua and Barbuda, The Bahamas, Barbados, Belize, Bermuda, British Virgin Islands, Cayman Islands, Cuba, Dominica, Dominican Republic, French Guiana, Grenada, Guadeloupe, Guyana, Haiti, Jamaica, Martinique, Montserrat, Netherland Antilles (Aruba, Bonaire, Curaçao, Saba, St. Eustatius, and the Dutch half of St. Martin [Sint Maarten]), St. Kitts and Nevis, St. Lucia, St. Vincent and the Grenadines, Suriname, Trinidad and Tobago, and Turks and Caicos Islands.

The U.S. Caribbean territories, Puerto Rico and the U.S. Virgin Islands, were not included as part of the region under investigation.
